# Correlation of Lack of Fusion Pores with Stress Corrosion Cracking Susceptibility of L-PBF 316L: Effect of Surface Residual Stresses

**DOI:** 10.3390/ma15207151

**Published:** 2022-10-14

**Authors:** Arshad Yazdanpanah, Mattia Franceschi, Pietro Rebesan, Manuele Dabalà

**Affiliations:** 1 Department of Industrial Engineering, University of Padova, Via Marzolo 9, 35131 Padova, Italy; 2 National Institute for Nuclear Physics (INFN), Padova Division, Via Marzolo 8, 35131 Padova, Italy

**Keywords:** additive manufacturing, laser powder bed fusion, stress corrosion cracking, lack of fusion porosity, localized corrosion, residual stress, machining

## Abstract

Stress corrosion cracking (SCC) of laser powder bed fusion-fabricated 316L was studied under the variation in energy input density to emulate the existence of distinctive types of defects. Various electrochemical polarization measurements were performed in as-received polished and ground states, to elucidate the effect of defect type on corrosion and SCC behaviour in marine solution. The results revealed severe localized corrosion attack and SCC initiation for specimens with a lack of fusion pores (LOF). Moreover, the morphology of SCC was different, highlighting a more dominant effect of selective dissolution of the subgrain matrix for gas porosities and a more pronounced effect of brittle fracture at laser track boundaries for the specimens with LOF pores.

## 1. Introduction

Metal additive manufacturing (MAM) is an emerging technology, gradually substituting conventional production methods, due to the flexibility in design, lower material loss and energy consumption, and significantly lower production time, notably for complex geometries [1]. Laser powder bed fusion (L-PBF) is recognized as one of the most widely developed MAM methods, relying on layer-by-layer production of almost-full-density parts, employing a high-energy laser beam [2]. The inherent L-PBF process features, such as highly localized laser beam and extremely rapid melting/solidification of the powder, consequently, lead to the unique nonequilibrium microstructure of the as-received components. The submicron cellular/columnar structure combined with the formation of nonequilibrium phases, enriched in certain alloying elements, are the common features of the L-PBF method [1,2,3,4]. Additionally, such inherent solidification characteristics are believed to eliminate the formation of common unwanted phases present in conventionally produced materials, such as manganese sulphide (MnS) in austenitic stainless steels, which could significantly improve the localized corrosion behaviour [1,5,6,7]. However, it is common knowledge that process parameters (e.g., laser power, scan speed, hatch spacing, and layer thickness) and material properties (e.g., powder size distribution, specific heat, and thermal conductivity) substantially affect the final microstructure and type of the defects present in L-PBF-processed components [1]. Depending on the mentioned parameters, gas porosities, lack of fusion (LOF) pores, keyhole defects, and various other defect types could be found that noticeably affect the mechanical properties (under constant or cyclic loading) and corrosion behaviour of the L-PBF parts [8].

Among all types of defects, LOF pores have been identified by various authors as one of the most critical defect types, as a consequence of their bigger size and irregular shape, which is believed to considerably alter the corrosion characteristics of MAM-processed parts [9]. For instance, in an investigation on direct-energy-deposited alloy 304 by Melia et al. [7], they reported a dramatic decrease in breakdown potential and activation of LOF pores as crevice sites, emphasizing noticeably lower localized corrosion resistance. Schaller and co-authors [10] reported the same preferential corrosion incidence in LOF sites due to geometrical reasons and substantially higher pitting potential in areas without the presence of pores for L-PBF 304. Moreover, Sander et al. [11] highlighted the reduced sensitivity in localized corrosion resistance to slight variations in pore size for L-PBF 316L within the range of the full-density condition. A recent investigation by Laleh et al. [12], on three- and two-dimensional characterization of LOF pores after corrosion attack, emphasized that the type and the shape of the pores present on L-PBF specimens are the main factors in risk assessment of L-PBF 316L to localized corrosion attack, suggesting a detrimental effect of LOF pores due to their inherent irregular shape. In another study on noise analysis of L-PBF 316L by Zhang et al. [13], they emphasized that with an increase in both pore density and size, pit initiation rate and growth probability increased substantially, leading to higher susceptibility of the material to localized corrosion attack.

The surface quality of MAM processes, which is the key factor for almost all industrial applications, has received much attention in the past decade and is believed to be greatly associated with the appropriate choice of process parameters [1,2,14]. Despite extensive improvements in process parameter optimization for attaining higher surface quality, there is still a necessity for post-processing treatments of the surface, such as selective laser remelting, shot peening, sand blasting, grinding, etc. Among all these methods, grinding has been the most precise and controlled method that is widely used for the enhancement of surface quality and attainment of required precise geometrical/dimensional tolerances. Nevertheless, surface grinding introduces heavy plastic deformation, microstructural alterations, strain-induced phase transformations, and high magnitudes of residual stress (RS) on the surface [15,16,17]. It is well established that machining methods affect the corrosion behaviour and stress corrosion cracking (SCC) susceptibility of industrial components (manufactured by conventional methods) and there is still considerable controversy surrounding the direct individual effect of machining-induced surface RS on SCC initiation susceptibility and no general agreement exists on whether RS induced by machining could solely cause SCC initiation [16,17,18,19,20,21,22]. Such contradictions have generally been ascribed to the microstructural variations (e.g., alloy type, grain size, constituent phase percentage), type of manufacturing processes (e.g., hot/cold rolling, extrusion, casting, and MAM) for the raw material, applied test method (e.g., slow strain rate testing, constant loading/straining, bending, immersion, and cyclic loading), and environment (e.g., acidic, alkaline, saline, and boiling magnesium chloride) used for SCC studies in various investigations.

Despite the importance of SCC in demanding applications (e.g., oil and gas, power, and nuclear industries) and the catastrophic nature of such failures, to the best of our knowledge, there has been limited discussion on SCC susceptibility of L-PBF fabricated parts, while SCC risk assessment of LOF pores has never been dealt with and the mechanisms responsible for the SCC initiation for the specific case of LOF pores have not yet been established. In the authors’ previous investigations [23,24], an attempt was made to systematically assess the effect of surface tensile RS on the SCC behaviour of L-PBF-processed 316L in the range of process parameters chosen for having almost-full-density specimens. Authors revealed that inherent microstructure, pore type, size, and distribution have a significant impact on SCC susceptibility of L-PBF 316L and the mechanism responsible for SCC initiation was hypothesized as the synergistic effect of selective dissolution of subgrain matrix, leaving the subgrain boundaries intact, combined with the mechanical rupture of the remaining bonds between the adjacent subgrains. Furthermore, Lou et al. [25] investigated the effect of post-processing heat treatments on the SCC propagation behaviour of L-PBF 316L in a boiling water reactor (BWR) environment and indicated the importance of high-temperature heat treatments on SCC morphology. Moreover, a recent investigation by Cruz et al. [26] on the SCC behaviour of L-PBF 316L, utilizing the slow strain rate method, confirmed cellular dissolution as the dominant mechanism in crack propagation. In the light of recent limited investigations on the SCC susceptibility of L-PBF-processed materials, concerns have been raised that call into question whether other types of defects present in MAM-processed components, such as LOF pores, could dramatically alter the SCC resistance of the material compared to the almost-full-density case.

This paper seeks to address the effect of LOF pores on the SCC initiation susceptibility of L-PBF 316L in marine solution. The selection of the environment followed the actual use case of austenitic stainless steels in industries located mostly in coastal regions (oil and gas industries, nuclear power plants, etc.), which provides valuable information without significantly altering the chloride ion contamination level, such as used in ASTM G36 (immersion in saturated boiling magnesium chloride solution). Specimens were fabricated under various energy input densities to intentionally introduce several types of defects. Various electrochemical polarization experiments were performed for in-depth analysis of variations in corrosion and SCC characteristics between the specimens in both as-received polished and ground states. This approach is notably important for enlightening the effect of LOF on SCC initiation under the individual effect of grinding-induced tensile stresses.

## 2. Materials and Methods

### 2.1. L-PBF Fabrication and Sample Preparation

Gas atomized powder of stainless-steel AISI 316L with a size distribution of 15–53 µm was used for the fabrication of cubic specimens with dimensions of 10 × 30 × 10 mm utilizing EOS M100 (EOS GmbH, Krailling, Germany) selective laser melting machine, under high-purity argon inert gas atmosphere (oxygen level less than 0.1%). The fabrication was performed on a stainless-steel AISI 304L platform preheated at 80 °C. Process parameters were intentionally chosen to have various types of porosities based on the authors’ previous investigations [23,24]. A bidirectional scan strategy with a rotational angle of 67 degrees between each layer and a stripe width of 5 mm was employed. The layer thickness and hatch spacing were maintained at 20 µm and 70 µm, respectively. Process parameter variation for each specimen with the corresponding volumetric energy input density is listed in Table 1. The volumetric energy input E (J·mm^−3^) is calculated as E = P·(h·v·t)^−1^, where P is the laser power (W), v is the laser scan speed (mm·s^−1^), t is the layer thickness (mm), and h is the hatching spacing (mm).

In order to increase the repeatability and accuracy of the experimental corrosion results, and to isolate the microstructural characteristics from the possible near-surface (last few built layers) microstructural alterations, one millimetre was removed from the last built layer of all L-PBF-fabricated samples utilizing electro discharge machining (EDM) method. Specimens with various energy input levels were analysed in two conditions, the as-received polished and ground state. To isolate the microstructural effects (defect type and size) on corrosion characteristics from the surface quality, one group of specimens was polished according to standard metallographic sample preparation up to 0.4 µm silica colloidal suspension. Samples were then washed ultrasonically for 20 min in 10% ethanol solution in distilled water to remove the excess impurities trapped in pore sites. Specimens were then rinsed with pure ethanol and dried in a low-pressure vacuum chamber for 30 min and kept untouched for 72 h in air at room temperature, to have stable native oxide layer formation on the surface. Another set of samples was ground utilizing a flat grinding machine, keeping constant the rotational speed of the grinding wheel (grit size of 80 µm) at 5000 rpm and the depth of cut at 20 µm using a coolant solution. With this approach, the surface residual stress was isolated from other grinding-induced surface modifications (as a result of identical surface roughness, the thickness of machining-induced microstructural alterations, and strain-induced phase transformations). Thus, the synergistic effect of grinding-induced tensile RS on the SCC behaviour of distinct types of defects was isolated. Ground specimens were also washed ultrasonically, rinsed, dried in a low-pressure vacuum chamber, and kept untouched for 72 h under identical conditions applied for as-received polished specimens.

### 2.2. Residual Stress Measurement

RS measurements were performed on the surface of the specimens prepared in both conditions of the as-received polished and ground state. Spider TM X GNR X-ray diffraction unit (GNR S.r.l. Analytical instrument group, Agrate Conturbia (NO), Italy) with a radiation source of chromium, kβ filter of vanadium, a spot size of 1 mm, working at 30 kV, and 90 µA was employed for RS measurements. The {311} plane (as the austenitic peak) was chosen for the diffraction peak implementing the sin^2^ (ψ) method with variation of 2θ angle between 0 and 43 degrees performing the measurements at 9 different angles. Acquired diffraction patterns were evaluated utilizing STRESS.NET software, provided by the manufacturer of the equipment, for the calculation of RS magnitude. RS was measured in the longitudinal direction, corresponding to length of 30 mm, and transverse direction, perpendicular to the grinding direction for ground specimens, to have a clear idea regarding the RS magnitude and state in various directions.

### 2.3. Electrochemical Polarization and SCC Experiments

To elucidate the effect of defect type and grinding-induced RS on corrosion and SCC behaviour, various electrochemical polarization methods were utilized on both groups of as-received polished and ground specimens. All the experiments were conducted in 3.5% sodium chloride solution in distilled, deionized aqueous solution to emulate the real environment in coastal regions with mild contamination of chloride ions, according to standard test methods reported by ASTM G59. The surface area in contact with the solution was a circle with a diameter of 6 mm and the standard three-electrode method was implemented with the standard calomel electrode (SCE) as the reference, a platinum cage as the counter, and the specimen as the working electrode utilizing Amel 2549 potentiostat unit (Amel Electrochemistry S.r.l., Milan, Italy). All electrochemical polarization experiments were performed after keeping the specimens in contact with the solution for 30 min prior to the experiments, to have the same condition after equilibrating with the solution.

Potentiodynamic polarization measurements were conducted in a range of −250–1000 mV with respect to open circuit potential measured prior to the start of the test (measured for 30 min). Galvanostatic experiments were performed at a current density corresponding to 10% higher than the breakdown current density measured from potentiodynamic polarization experiments. The galvanostatic method is a viable method to have accelerated corrosion studies without dramatically altering the chloride ion concentration, which could provide tremendous information regarding the passive layer behaviour over time of exposure at a constant current density of critical pitting/SCC. Thus, a polarization experiment under constant current density could potentially isolate the microstructural effects on corrosion and SCC characteristics from other possible electrochemical parameters involved in the electrochemistry of the test setup.

### 2.4. Microstructural and SCC Characterization

Microstructural analysis was conducted before and after polarization experiments for both as-received polished and ground specimens. Porosity analysis was performed by taking 15 pictures with magnification levels of 5 to 20 X utilizing a Leica DMRE (Leica Microsystems S.r.l., Milan, Italy) optical microscope. With the aim of clarifying the underlying microstructure, polished specimens were electro-etched in 10% oxalic acid solution in ethanol at 6 V for 20 to 30 s. Furthermore, the surface and cross-section of the specimens were examined after galvanostatic polarization experiments to reveal the effect of size and type of observed defects on corrosion and SCC behaviour. A field emission gun scanning electron microscope (FEG-SEM) Zeiss ULTRA 55 (Carl Zeiss NV, Zaventem, Belgium) and Leica Cambridge LEO STEREOSCAN 440 (Leica Microsystems S.r.l., Milan, Italy) scanning electron microscope (SEM) with an accelerating voltage of 15 KeV were employed for in-depth analysis of the fractographic features.

## 3. Results

### 3.1. Porosity and Microstructural Characterization

**Figure 1:**
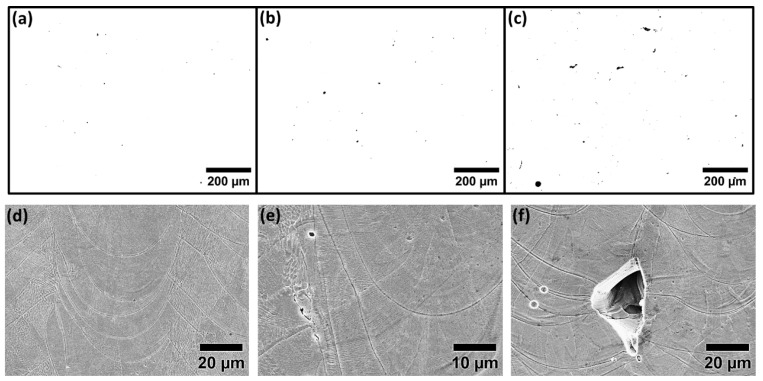
Optical and SEM microstructural analysis of L-PBF specimens of (**a**,**d**) group one, (**b**,**e**) group two, and (**c**,**f**) group three.

**Figure 2:**
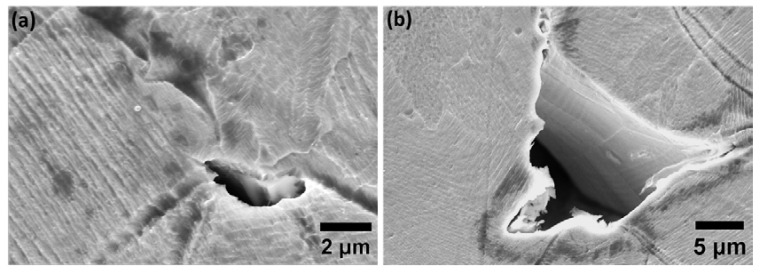
(**a**,**b**) SEM microstructural analysis of the LOF pores present in the specimens of group three.

**Figure 3:**
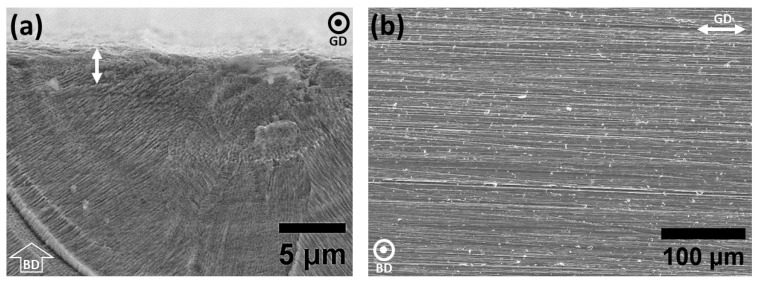
(**a**) Grinding-induced plastically deformed layer thickness and (**b**) surface of the ground specimens highlighting the machining marks. (BD) Build direction, (GD) grinding direction.

Microstructural analysis of specimens with various energy input levels was conducted to have a clear idea regarding the shape and size of the defects present on the surface before further in-depth electrochemical analysis. Porosity analysis of the specimens with various energy input densities is summarized in Table 2. As shown in Figure 1a,d, specimens in group one show the highest density with a few signs of small spherical pores and are considered as the representative of the highest quality that could be obtained by the L-PBF process, considering density and size distribution of defects. For the case of group two, a gradual increase in pore size as a consequence of higher laser power is observed (Figure 1b,e) and for group three (Figure 1c,f), the transition of the pores from gas porosities to LOF pores is detected as a consequence of higher scan speed and lower laser power during the L-PBF fabrication. More in-depth analysis of the specimens in group three (Figure 2) highlights the presence of LOF pores, primarily at the melt pool boundaries along with the adjacent laser track boundaries. However, as shown in Figure 1f and Figure 2, in all cases, LOF pores have irregular shapes with signs of entrapped powder particles inside. Such entrapped particles could potentially alter the density measurements with conventional Archimedes methods. Moreover, microstructural analysis of the etched surface of the specimens (Figure 1d–f) highlights the existence of columnar/cellular subgrain structure in addition to directional solidification along the build direction of the specimens. The general microstructure of all groups was fully austenitic and the presence of grains continued along several build layers was also frequently detected. The cross-sectional analysis of the ground surface was also performed to detect alterations in the thickness of the deformed layer on the surface. Obtained results emphasized identical thickness for all cases and, for the sake of simplicity, the grinding-induced deformed layer is shown merely for the specimens in group one with the highest density (Figure 3).

### 3.2. Residual Stress Analysis

RS measurements were conducted on the surface of the specimens for both as-received polished and ground states in both longitudinal (along the machining direction) and transverse (perpendicular to the machining direction) directions, with the results presented in Table 3. Specimens in the as-received polished state (after keeping untouched for 72 h) showed compressive RS in both longitudinal and transverse directions, emphasizing slight anisotropy in RS magnitude depending on the direction of the measurement. However, experimental results underlined the dependency of the RS magnitude on energy input density, indicating slightly higher compressive RS magnitude for specimens with higher energy input density. In contrast, RS measurement data for the ground specimens showed the dominancy of the effect of grinding on the RS state of the specimens, highlighting the presence of nearly identical tensile RS state and magnitude on the surface of all specimens, regardless of their energy input density.

### 3.3. Electrochemical Polarization Results

**Figure 4:**
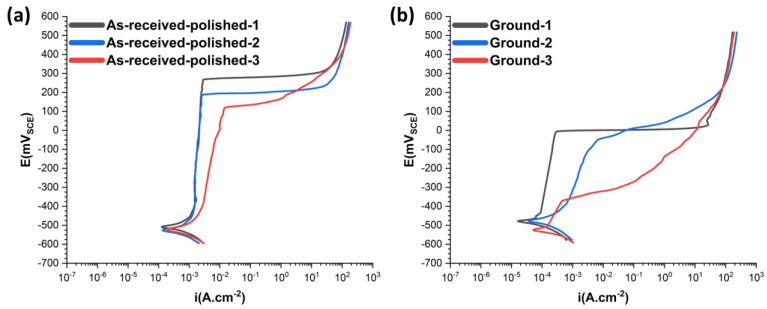
Potentiodynamic polarization results in specimens with various energy input densities, for (**a**) as-received polished and (**b**) ground state.

Electrochemical polarization experiments were performed to explicate the effect of inherent defects of the L-PBF process on corrosion properties and SCC initiation susceptibility. Potentiodynamic polarization results, as shown in Figure 4, emphasize the reduced sensitivity of the corrosion potential (E_corr_) and corrosion current density (I_corr_) on the defect type. However, there exists a profound correlation between breakdown potential (E_break_) and breakdown current density (I_break_) with energy input density. Specimens with the highest density indicated the highest E_break_ and lowest I_break_ compared to other specimens. With the decrease in the density of the specimens (group two), E_break_ decreased slightly by 85 mV compared to the full-density, group one, specimens. However, I_break_ was almost identical for both groups one and two, highlighting the reduced sensitivity of I_break_ to slight variations in pore density under identical pore type. With the transition of the pores type from small gas porosities to uneven-shaped LOF pores, detected in specimens in group three, a substantial decrease in E_break_ and increase in I_break_ are noted for the as-received polished specimens (Figure 4a), emphasising the strong correlation of pore type with the potentiodynamic polarization results. E_break_ decreased from 270 mV for the as-received polished specimens in group one to 170 mV for the specimens in group three, owing to the presence of LOF pores. Additionally, I_break_ increased considerably from 0.0028 A.cm^−2^ for the specimens with full density (group one) to 0.013 A.cm^−2^ for the samples in group three, indicating almost five-times higher current density.

Furthermore, for the ground case, all specimens, regardless of energy input level (Figure 4b), show considerably lower E_break_ compared to the as-received polished state. Specimens in group one (with the highest density) indicated relatively higher E_break_ compared to the other two groups with a value of −7 mV. Nevertheless, the difference between group one and group two (in which the type of defects is identical and only the size and distribution of gas porosities differs) was generally related to the I_break_ more readily than the E_break_, revealing a higher dependency of the corrosion severity to the pore surface area under tensile-grinding-induced RS. Remarkably, specimens in group three revealed the strong effect of surface RS by demonstrating a considerable reduction in the transpassive zone range. Ground specimens in group one maintained a transpassive state for a range of 433 mV, which is significantly higher than the range of transpassive state for group three, which was 127 mV, emphasizing the drastically narrower range of transpassive zone in the case of the presence of LOF pores under the effect of machining-induced surface RS.

**Figure 5:**
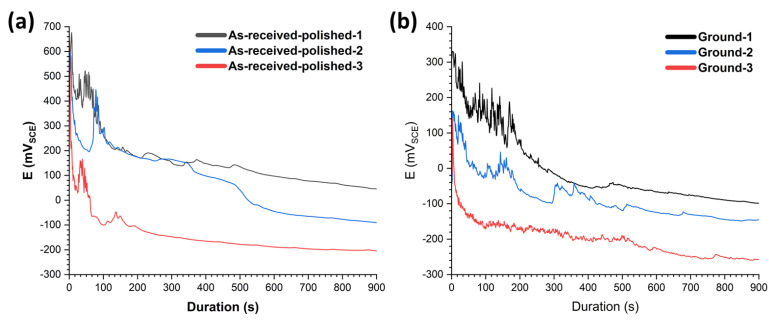
Galvanostatic polarization results in specimens with various energy input densities, for (**a**) as-received polished and (**b**) ground state.

Galvanostatic polarization measurements were performed to shed light on the influence of pore type and size on passive layer evolution over time of exposure (under constant current density), which is a viable method for accelerated corrosion and SCC studies. Acquired results for the case of as-received polished specimens, as shown in Figure 5a, emphasize higher peak potential for specimens in groups one and two, maintaining comparable perturbations in potential during the early stages of the measurements. For the case of the specimens in group three, the peak potential was noticeably lower, suggesting less resistance in the passive layer to charge transfer. Likewise, after the breakdown of the passive layer, indicated by the potential decrease, as shown in Figure 5a, the potential shows a meaningful correlation with the type and size of the pores. For the specimens in group three, the steady-state potential was lower compared to groups one and two. Even though the potential followed almost identical behaviour during the first period after the sudden potential drop (for the specimens in groups one and two), a slight decrease was detected for the specimens in group two after 400 s. Additionally, the situation for the ground specimens, as shown in Figure 5b, shows a more critical condition, indicating lower peak potential and higher perturbations during the initial stages of the polarization experiments (compared to the as-received polished case). Specimens in groups one and two highlighted longer duration at peak potential compared to group three, suggesting low resistance in the passive layer for the specimens in group three. While higher instabilities were detected for specimens in groups one and two for the ground specimens, the potential at the steady-state region followed the same trend with the variation in energy input level. Additionally, the potential perturbations were less evident for the specimens in group three, owing to the ease of passive layer breakdown compared to the other specimens with fewer pores (groups one and two). The most noticeable observations to emerge from the electrochemical polarization experiments were first, the significantly lower resistance of the passive layer for the specimens with LOF pores, and second, the less noticeable difference between specimens with the same type of pores (through breakdown potential of potentiodynamic and less difference in peak potential in galvanostatic polarization measurements) with a slight difference in pore size. However, the perturbation in potential during the entire duration of the galvanostatic measurements was considerably greater for all cases at the ground state compared to the as-received polished case.

### 3.4. Microstructural Analysis of SCC and Localized Corrosion Defects

**Figure 6:**
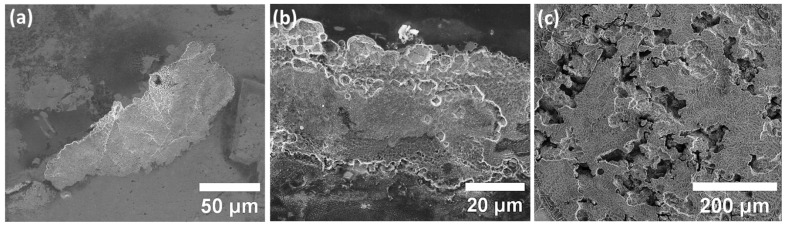
SEM microstructural analysis of the corroded surface of specimens of as-received polished state of (**a**) group one, (**b**) group two, and (**c**) group three highlighting the morphology of corrosion attack.

Microstructural analysis was performed on specimens in all groups after the galvanostatic polarization measurements for in-depth analysis of the corrosion defect type and to enlighten the possible correlation between the observed electrochemical events with the microstructural characteristics. Surface SEM analysis of the specimens in the as-received polished state, as presented in Figure 6, highlights the major difference between the specimens of various groups. Specimens in groups one and two exhibit indications of selective dissolution of the subgrain matrix and pitting corrosion as the dominant corrosion defect type (Figure 6a,b). However, the difference between specimens in groups one and two was restricted to the slightly more severe condition for the samples in group two. The corrosion attack on the surface of both groups one and two was localized and limited to small islands on the surface. For the case of group three, the surface was rigorously corroded both on the surface and depth, emphasizing a drastically higher corrosion attack compared to the specimens in other groups.

**Figure 7:**
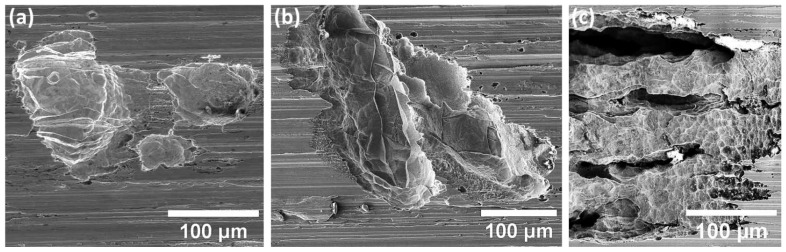
SEM microstructural analysis of the corroded surface of ground specimens of (**a**) group one, (**b**) group two, and (**c**) group three highlighting the morphology of SCC initiation and corrosion characteristics.

Additionally, surface SEM analysis of the ground specimens, as shown in Figure 7, uncovers a substantial shift in the corrosion behaviour compared to the as-received polished case. For the case of specimens in groups one and two (Figure 7a,b), the corrosion defect type shifts from selective dissolution, seen in as-received polished specimens, to highly localized pitting and SCC. SCC was detected in the base of the pits for both groups through slightly higher branching for the specimens in group two. Cracks were detected mostly at the laser tracks and melt pool boundaries for both groups one and two. Remarkably, over exposure time, corrosion attack emphasized higher dependency to persist on the surface rather than an increase in pit depth for the existing pits. However, the situation markedly changes for the specimens in group three (Figure 7c), highlighting the severe corrosion attack in highly localized places, which were LOF pore sites. Pitting and crevice corrosion were the major corrosion types that were identified for specimens in group three suggesting the higher dependency of LOF pores to localized corrosion attack rather than SCC initiation, as observed previously in specimens in groups one and two.

**Figure 8:**
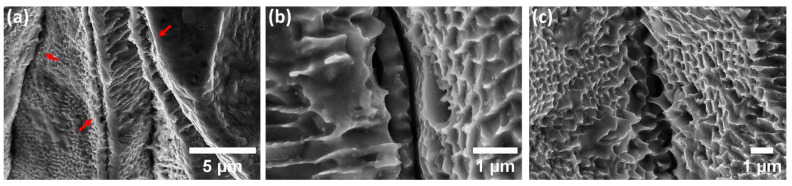
(**a**–**c**) High-resolution FEG-SEM images of the specimens in groups one and two highlighting the SCC morphology (shown by red arrows in (**a**)).

Further evaluation of the specimens in groups one and two with higher magnification FEG-SEM, as shown in Figure 8, reveals the selective dissolution of the subgrain matrix in the close vicinity of the crack initiation sites, leaving the boundaries of subgrains intact. Furthermore, it is apparent that the laser track boundaries between adjacent laser passes and melt pool boundaries are the most susceptible sites for SCC initiation (Figure 8a).

**Figure 9:**
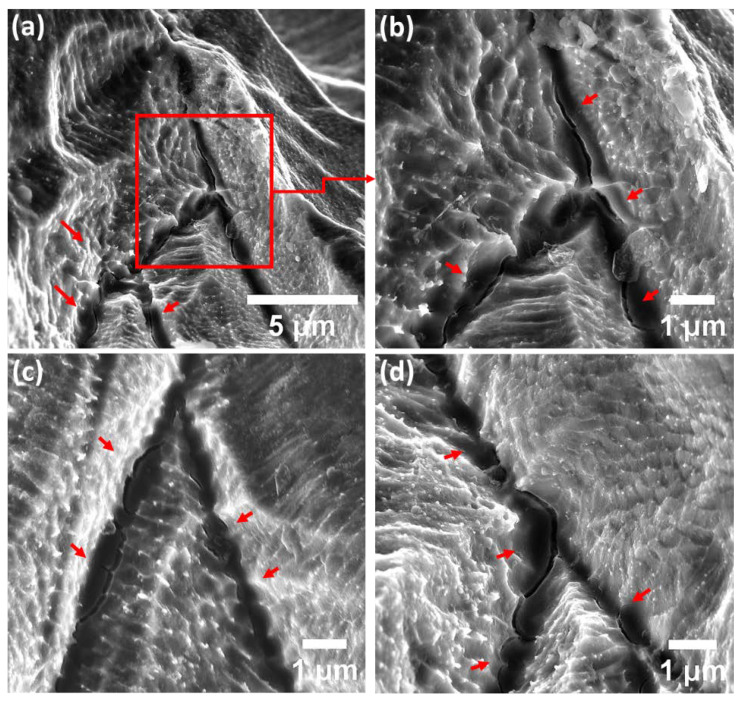
(**a**–**d**) High magnification of the SCC morphology (shown by red arrows) for the specimens in group three with the presence of LOF pores.

Additionally, high-magnification SEM analysis of the ground specimens in group three, which comprised LOF pores, revealed underlying localized corrosion and SCC initiation behaviour. As shown in Figure 9, even though cracks are generally initiated at the interface of adjacent melt pool boundaries and laser tracks (as is the case for specimens in groups one and two consisting of gas pores), the morphology of cracks is different from the type detected previously for the specimens with gas porosity defects. Detected cracks were mainly brittle with sharp edges, which emphasized the predominant influence of mechanical rupture compared to corrosion-induced weakening of the subgrain structure. Nevertheless, it is worth clarifying that the dominant observed defect type for the specimens with LOF pores was severe pitting and crevice corrosion and the detected cracks were limited to the zones near the ground surface.

## 4. Discussion

### 4.1. Microstructural Characteristics of L-PBF

The presence of several defect types is believed to be an indivisible part of the L-PBF process and, in general, MAM methods [9], which is recognized to be greatly determined by the shape and size of the powder particles and L-PBF process parameters, commonly known as energy input density [4,27]. During the past decade, extensive research has been conducted on process parameter optimization and the correlation of process parameters with detected defect types. It is generally accepted that with an increase in energy input density, there exists a transition from LOF to gas porosities and under excessive circumstances, keyhole defects could form during the L-PBF fabrication [28,29,30]. However, short-range variation in any of the parameters affecting the energy input density (e.g., laser power, scan speed, hatch spacing, and layer thickness) could lead to slight variations in the size and distribution of the pores, without significant alteration in the defect type [29,31]. Additionally, the intrinsic microstructure of the L-PBF fabrication method could be analysed at the micro and submicron levels. The microstructure of the L-PBF method comprises melt pools and melt pool boundaries and depending on process parameters, scan strategy, and the characteristics of the laser source, the interaction of the melt pools between neighbouring layers could significantly vary, which could result in macro precipitation/segregation of certain alloying elements. However, it is generally accepted that with the right choice of process parameters, such elemental anisotropies in macro level could be reduced through extremely rapid thermal cycles in the L-PBF process [8,32]. The EDX line scan analysis of the specimens from all groups (not presented herein) confirmed the elimination of macro-elemental precipitation/segregation in the current investigation as well. However, at the submicron scale, cellular/columnar structure is the common microstructural feature in the L-PBF and, in general, MAM-processed parts. The extremely rapid solidification and constitutional undercooling are believed to be responsible for the formation of nonequilibrium phases at subgrain boundaries [33]. It is well established that subgrain boundaries are generally enriched in molybdenum and chromium for the case of L-PBF-fabricated stainless steels [14,34], highlighting the submicron precipitation/segregation of alloying elements at subgrain level.

### 4.2. Influence of Defect Type on Electrochemical Polarization Response

As-received polished specimens with a variety of energy input densities were analysed by electrochemical polarization techniques to shed light on the effect of defect type on corrosion characteristics of L-PBF-processed 316L austenitic stainless steel. To fully understand the correlation of defect type and size with corrosion behaviour, a few factors must be explained. It is well indicated that one of the main active sites for localized corrosion attacks, such as pitting and/or SCC, in conventional stainless steels is the presence of MnS particles [5,6,35]. It has been reported by various research groups that extreme thermal cycles during the L-PBF process eliminates the formation of such phases that could considerably enhance the localized corrosion and SCC resistance of L-PBF compared to conventional counterparts [36,37,38]. In addition, it is well recognized that surface roughness plays a crucial role in localized corrosion resistance of alloys with passive behaviour. Since in the current investigation, specimens were polished (for the as-received polished specimens), it could be stated that the effect of surface roughness on the electrochemical behaviour was also diminished. Thus, variation in pore type and size due to the variations in energy input density could be considered as the dominant factor responsible for the observed variations in corrosion characteristics. As seen previously, potentiodynamic polarization results highlighted a slight reduction in breakdown potential with an increase in the pore size. Given that the dominant pore shape was spherical for both groups and the difference was the size of defects, such an observation could be justified based on an extension to the differential aeration hypothesis (DAH) introduced by Evans et al. [39] and further developed by Macdonald et al. [40,41,42]. The DAH states that the local anode and cathode are spatially separated, with the anode being in the cavities, owing to less access to cathodic reactants, such as oxygen for geometrical reasons, and the surface in direct contact with the solution becoming the cathode, which is forming a coupled environment. It is hypothesized and later examined extensively [39,40,41,42,43,44] that in order to compensate the positive charge caused by metal dissolution reactions occurring in the cavities, the negatively charged chloride ions are transported into the cavities, leading to a reaction between the chloride ions and the positive hydrogen charges produced by the hydrolysis of the metal cations. Such reactions significantly decrease the pH level in local sites of cavities. Such an increase in the acidity of the solution leads to the annihilation of the ability of the passive layer present in cavities to repassivate, leading to severe localized corrosion and/or SCC attack in such sites. What we observed in our recent investigations on the SCC initiation behaviour of L-PBF 718 nickel-iron-based superalloy [45], and also LPBF 316L under the short-range variation in energy input density [23,24], was that when the L-PBF-fabricated part was full density with small spherical pores, minor changes in pore size (by means of pore surface area) did not drastically change the corrosion behaviour. A recent study by Sander et al. [31,46] also emphasized that even with an increase in chloride ion contamination level, small pores were not activating as active localized corrosion sites. Keeping in mind the DAH, the mentioned inconsistency could be explained by the microstructural characteristics of L-PBF, which is quite different from the conventional case. As explained in previous sections, microstructure of L-PBF and, in general, MAM consists of cellular/columnar subgrains with a dimension of a few hundred nanometres. Owing to the formation of nonequilibrium phases during the rapid solidification of each melt pool, compositional variations exist between the subgrain matrix and the subgrain boundaries, leading to formation of submicron-level coupling between the subgrain matrix and the boundaries. Thus, the distance between the local anode and cathode dictated by DA hypothesis is in the submicron level. On the other hand, in the case of the presence of small spherical pores on the surface, DAH dictates that the pore interior will function as the anode and the surface acts as a cathode. However, since the distance between the local anode and cathode in the case of small pores is much higher compared to submicron coupling, considering the Ohm’s law (which states that the electrons preferentially move in the shortest distance from anode to cathode), it could be hypothesized that the coupling between the subgrain boundary and matrix forms rather than the coupling between the existing pores and the adjacent surface. Thus, the surface of L-PBF itself could be separated into thousands of small, coupled environments, consistent with DAH principles. However, it is worth mentioning that the current justification is only valid for the small spherical pores. As a result, it could be clearly stated from the findings of the current investigation that there exists a critical pore size (for spherical gas porosities), wherein lower than that, the pore is not activated as a preferential site for localized corrosion and/or SCC initiation. As previously shown in Figure 6, for the case of specimens in groups one and two, for which the dominant defect type was gas porosities, the common observed corrosion defect (for the as-received polished state) was the selective dissolution of the subgrain matrix, leaving the subgrains undamaged. Notwithstanding, for the case of specimens with LOF pores, the condition is readily available for the strong coupling between the LOF pores and the surface, leading to a substantial increase in the acidity of the LOF cavities, resulting in the detected severe localized corrosion attack at LOF pore sites.

### 4.3. Influence of Pores Size and Type on SCC Initiation under the Effect of Surface RS

It was established by Choudhary et al. [47,48] that the passive layer present on the surface of stainless steels is a dense layer of chromium oxide and repassivation is associated with the flux of chromium cations, emphasizing an enhancement in the passive layer stability with an increase in the flux of cations. On the other hand, based on the point defect model (PDM) developed by Macdonald et al. [49,50], the formation and annihilation of the passive layer are taking place concurrently and the noble behaviour of passive materials in corrosive environments is determined by the equilibrium between the formation and annihilation reactions. In the case of any variations in the environment, the mentioned equilibrium is disturbed, consequently leading to a higher kinetics of annihilation reactions, resulting in localized corrosion attack and/or SCC initiation. In the case of the presence of the LOF pores, based on DAH explained previously, the condition inside of the LOF pores (as a consequence of the irregular shape and size) is ideal for the substantial intensification in the acidity of the microcell formed in such local sites, which is also confirmed by Laleh et el. [12,51]. As a result, the balance between the formation and annihilation of the passive layer inside of LOF pores is disrupted, leading to faster kinetics of passive layer destruction compared to the formation and severe corrosion attack at such local sites of LOF pores. However, as can be observed in Figure 9, the dominant corrosion attack is still selective dissolution of the subgrain matrix, even inside the LOF pores.

A further noteworthy observation that needs more clarification is the morphology of the cracks in LOF pores. It is apparent from high-magnification SEM images (Figure 9) that the cracks emphasize quite different morphology compared to full-density specimens. Cracks were primarily brittle, while originating from the similar locations of laser tracks and melt pool boundaries. Such a sharp crack morphology could be clarified based on the synergistic effect of stress concentration and micro-electrochemical cell formation in LOF pore sites. Since the shape of the LOF pores is irregular, the stress concentration could considerably be higher compared to small gas porosities. On the other hand, the localized corrosion attack inside of LOF pores is more severe (as explained previously by DAH) and the condition is ideal for the formation of more sharp corners and edges at the sites of laser tracks and melt pool boundaries (by virtue of selective dissolution of the subgrain matrix near the melt pools, as shown in Figure 9). Therefore, the synergistic effect of a higher rate of selective dissolution of the matrix combined with an increase in stress intensity at laser track sites, consequently, leads to a superior tensile stress magnitude on such sites. On the other hand, it is believed that the nonequilibrium phases present at subgrain boundaries possess less ductility compared to the austenitic matrix of subgrains [33], which could effectively describe the brittle fracture of the melt pool and laser track boundaries between two adjacent layers. However, such a justification could be only effective for the cases in which the localized corrosion rate is high and the mechanical rupture is the dominant mechanism responsible for SCC formation.

## 5. Conclusions

This paper called into question whether the type of defects present in laser powder bed fusion-fabricated parts could alter the stress corrosion cracking initiation susceptibility of 316L austenitic stainless steels in marine environments. The effect of grinding-induced surface residual stresses was elucidated on the SCC performance of the parts with distinct energy input densities. The main conclusions can be summarized as follows:

- A strong correlation was identified between the electrochemical polarization response and defect type and size. For the case of a lack of fusion pores, the range of passivation was much narrower compared to the case of gas porosities.
- Machining-induced surface residual stresses directly affect the SCC initiation susceptibility and electrochemical polarization response of the samples, regardless of defect type.
- For the case of specimens with gas pores, with an increase in pore size, the material resistance to localized corrosion attack was slightly lower, highlighting the effect of pore size on corrosion behaviour.
- Ground specimens with the presence of a lack of fusion pores indicated severe localized corrosion attack at pore sites combined with the SCC at laser track boundaries near the surface.
- The morphology of cracks was different between specimens with gas porosities and lack of fusion pores, highlighting mostly brittle fracture at laser track boundaries for the specimens with lack of fusion pores and corrosion-assisted dissolution of subgrain matrix and fracture of the intact subgrain boundaries for the specimens with gas porosities.

## Figures and Tables

**Table 1:** Process parameters of L-PBF fabrication.

Sample Group No.	Laser Power (W)	Scan Speed (mm·s^−1^)	Energy Input (J·mm^−3^)
1	120	800	107
2	135	800	121
3	90	1200	54

**Table 2:** Porosity analysis of the specimens with various energy input density levels.

Sample Group	Density Mean (%)	Pore Surface Area Mean (µm^2^)	Max. Pore Surface Area (µm^2^)	Porosity Std Dev. (%)	Porosity Mean (%)
1	99.91	4.53	390.62	0.05	0.09
2	99.74	15.89	612.21	0.09	0.26
3	97.2	175.05	1843.83	0.5	2.8

**Table 3:** Residual stress magnitude measured with X-ray diffraction method for L-PBF 316L specimens in various groups.

Group No.	As-Received Polished		Ground	
	Longitudinal (MPa)	Transverse (MPa)	Longitudinal (MPa)	Transverse (Mpa)
1	−258 ± 6	−210 ± 8	308 ± 12	173 ± 9
2	−241 ± 9	−187 ± 12	332 ± 8	196 ± 9
3	−193 ± 9	−149 ± 6	314 ± 13	184 ± 12

## Data Availability

The raw data with the findings of this article cannot be shared since there is ongoing research on this topic.
